# Clinical comparison of single posterolateral plate with medial-cannulated-screw fixation and double-plate fixation for extra-articular distal humerus fractures

**DOI:** 10.1186/s10195-026-00918-6

**Published:** 2026-05-01

**Authors:** Hyoung-Seok Jung, Min-Su Chu, Jae-Sung Lee

**Affiliations:** 1 Department of Orthopedic Surgery, Chung-Ang University Gwangmyeong Hospital, Gwangmyeong, Republic of Korea; 2 Department of Orthopedic Surgery, Chung-Ang University Hospital, 224-1 Heukseok-Dong, Dongjak-Gu, Seoul, 156-755 Republic of Korea

**Keywords:** Double-plate, Single-plate, Medial screw, Distal humerus fracture, Ulnar nerve

## Abstract

**Background:**

Double-plate fixation is the gold standard for extra-articular distal humerus fractures, but it carries a substantial risk of postoperative ulnar neuropathy. Fixation using a single posterolateral plate with a medial cannulated screw may reduce ulnar neuropathy while maintaining fracture stability. This study aimed to compare the clinical outcomes of single-plate-with-medial-screw fixation versus double-plate fixation for extra-articular distal humerus fractures.

**Materials and methods:**

Fifty-six patients who underwent surgery for extra-articular distal humerus fractures (Arbeitsgemeinschaft für Osteosynthesefragen/Orthopaedic Trauma Association [AO/OTA] classification A2 or A3) between January 2018 and August 2024 were divided into a double-plate group and a single-plate-with-medial-screw group. We conducted a retrospective, nonrandomized comparative study. The double-plate fixation was used in 30 patients from January 2018 to October 2021, while the single-plate fixation with a medial screw was used in 26 patients from November 2021 to August 2024. All surgeries were performed using a posterior paratricipital approach. Bony union, radiographic healing, and loss of reduction were evaluated. Postoperative pain scores (visual analog scale at 2 days after the operation), operative time (minutes), elbow range of motion, elbow function (Mayo Elbow Performance Score [MEPS]), and the presence of postoperative ulnar neuropathy were compared between the two groups.

**Results:**

The double-plate fixation and single-plate fixation with a medial screw were performed in 30 and 26 patients, respectively. The mean age was 54.8 ± 19 (range, 17–85) years, and the mean follow-up duration was 18.2 ± 6.5 (range, 12–38) months. All fractures achieved solid osseous union at final follow-up. No significant differences were observed between the groups in terms of postoperative pain score, range of motion, and MEPS (all *p* > 0.05). However, the operative time was shorter for the single-plate-with-medial-screw group than that for the double-plate group (112.5 ± 25.7 versus 172.2 ± 35.2 min, *p* < 0.05), and the operative time was significantly associated with the fixation method (*p* < 0.05). In addition, postoperative ulnar neuropathy occurred less frequently with the single-plate-with-medial-screw group than with the double-plate group (8% versus 37%, *p* = 0.013).

**Conclusions:**

Both double-plate and single-plate-with-medial-screw fixation showed comparable union rates and functional outcomes in extra-articular distal humerus fractures. However, single-plate fixation with a medial screw required a shorter operative time and was associated with a lower incidence of postoperative ulnar neuropathy than double-plate fixation.

*Level of evidence* Level III, retrospective comparative study.

**Supplementary Information:**

The online version contains supplementary material available at 10.1186/s10195-026-00918-6.

## Introduction

Distal humerus fractures with transcondylar and intercondylar fractures can be challenging to treat due to complex anatomy, unique biomechanical forces, poor bone quality in older patients, and need for early range of motion [1–6]. These fractures typically result from high-energy trauma in younger patients, such as motor vehicle accidents or falls from a height, whereas in older patients with osteoporotic bone, they more commonly occur after low-energy falls [4]. Internal fixation with double plating is the current gold standard for these fractures; it provides excellent mechanical stability and has demonstrated favorable clinical outcomes in multiple studies [7, 8]. However, a notable concern associated with the traditional medial-plate placement is the risk of iatrogenic ulnar nerve irritation or neuropathy, which represents one of the most common postoperative complications in patients [9]. In addition, the medial plate tends to be prominent, particularly in patients with a low body mass index (BMI), and occasionally causes discomfort. As an alternative approach, the use of a posterolateral plate combined with a medial screw has emerged, aiming to maintain adequate fracture stability while potentially reducing the risk of postoperative ulnar neuropathy by avoiding extensive medial dissection and hardware placement [9, 10]. Although double-plating techniques have established robust biomechanical support and widespread clinical acceptance, there remains a paucity of comparative clinical studies directly evaluating outcomes—particularly nerve complications—between the standard double-plating and the alternative lateral-plating-with-medial-screw fixation. The existing literature lacks sufficient clinical data to validate whether a single posterolateral plate with medial-cannulated-screw fixation can achieve comparable outcomes in terms of fracture stability, union rates, and functional recovery. We hypothesize that medial-screw fixation combined with lateral plating may substantially reduce the incidence of ulnar neuropathy while achieving clinical and radiographic outcomes comparable to those of conventional double-plate techniques. Therefore, the purpose of study was to compare the outcomes of these two distinct fixation methods in adult patients with extra-articular distal humerus fractures.

## Materials and methods

This retrospective comparative study of extra-articular distal humerus fractures was approved by the institutional review boards of the two participating institutions. All patients who underwent surgery for extra-articular distal humerus fractures between January 2018 and August 2024 at two tertiary university hospitals were retrospectively reviewed. All patients had their diagnoses confirmed radiologically using X-rays and computed tomography imaging. Patients were included if they (1) had sustained extra-articular distal humerus fractures (Arbeitsgemeinschaft für Osteosynthesefragen/Orthopaedic Trauma Association [AO/OTA] classification A2 or A3), (2) had undergone open reduction and internal fixation, (3) were 18 years of age or older, and (4) had at least 1 year of follow-up. The exclusion criteria included previous surgery on the ipsilateral elbow, pathological fractures, open fractures with substantial soft-tissue loss, accompanying proximal forearm fractures, and inadequate follow-up or incomplete medical records. Patients were divided into two groups on the basis of the surgical fixation method: a double-plate group and a single-plate-with-medial-screw group. All patients were observed clinically and radiographically for at least 1 year.

### Surgical procedures

All surgeries were performed by two experienced elbow surgeons under either general anesthesia or a brachial plexus block. This study was conducted as a nonrandomized clinical trial. Double-plate fixation was used from January 2018 to October 2021, while single-plate fixation with a medial screw was used from November 2021 to August 2024. All patients underwent surgery in the lateral decubitus position. A nonsterile pneumatic tourniquet or a sterile tourniquet was applied as proximally as possible on the arm. The fracture was approached via a midline posterior incision using the paratricipital approach to provide adequate visualization of both columns of the distal humerus.

### Double-plate fixation

**Fig. 1:**
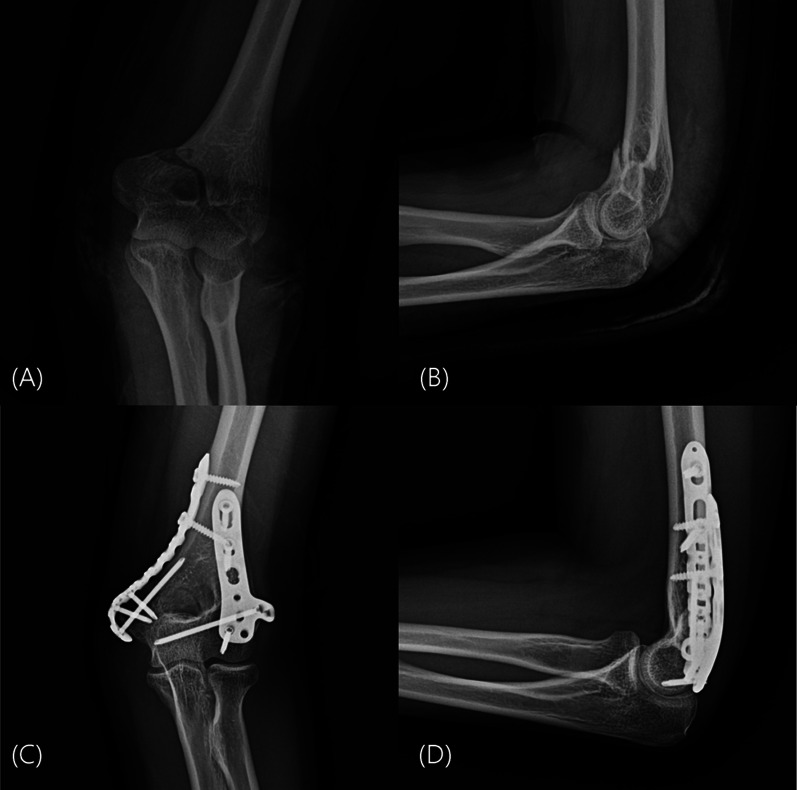
A 37-year-old female patient treated with double-plate fixation. **A**, **B** Preoperative radiographs showed an extra-articular distal humerus fracture. **C**, **D** Postoperative radiographs obtained at final follow-up demonstrated bony union

In the double-plate group, the ulnar nerve was first identified and released. Fracture reduction was then achieved through direct visualization and was temporarily held with a Kirschner wire (K-wire). Posterolateral plate fixation was performed using a variable-angle locking compression plate (VA-LCP) plate (DePuy Synthes, Inc.). A medial VA-LCP plate (DePuy Synthes, Inc.) was also applied to the medial column with protection of the ulnar nerve. Both plates were typically positioned in an orthogonal configuration (Fig. 1). Anterior subcutaneous transposition of the ulnar nerve was routinely performed during the final stage of surgery.

### Single-plate fixation with a medial screw

**Fig. 2:**
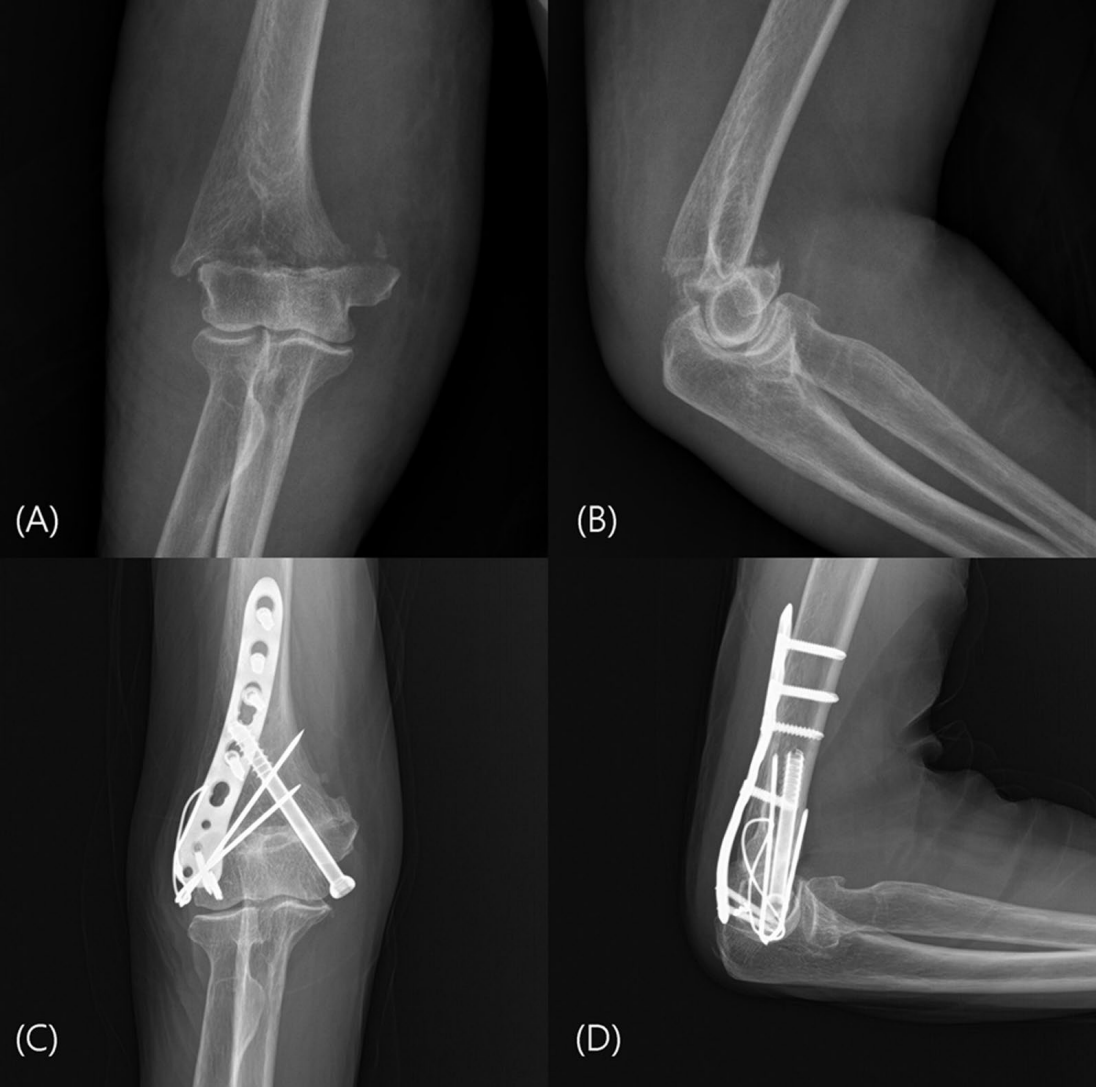
An 85-year-old female patient treated with single-plate fixation with a medial screw. **A**, **B** Preoperative radiographs showed an extra-articular distal humerus fracture with subsequent medial translation of the distal fragment. **C**, **D** Additional tension-band wiring was performed for lateral column stabilization. Postoperative radiographs obtained at final follow-up demonstrated bony union without any signs of displacement

**Fig. 3:**
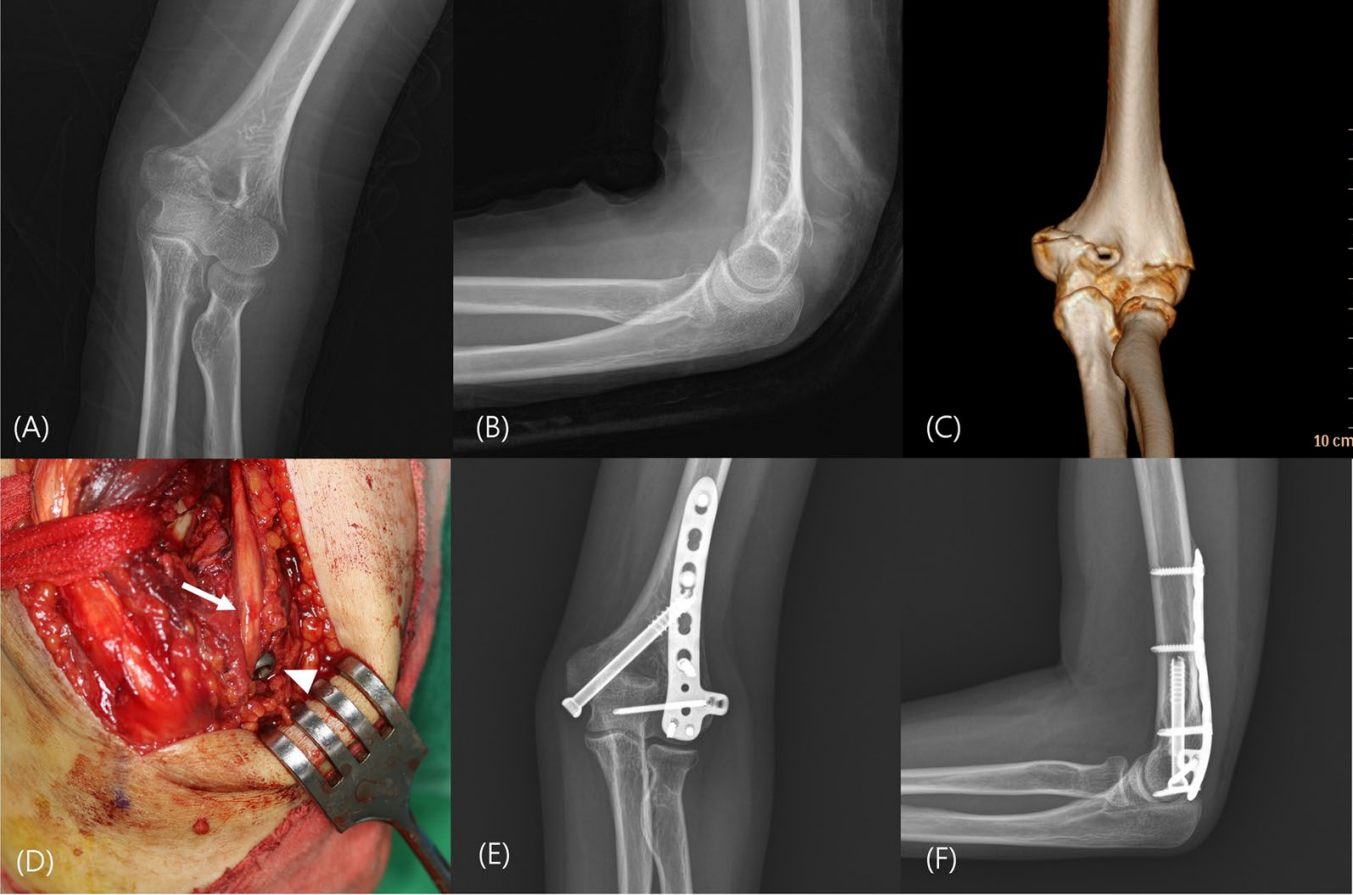
A 29-year-old female patient treated with single-plate fixation with a medial screw. **A**, **B** Preoperative radiographs showed an extra-articular distal humerus fracture. **C** Three-dimensional computed tomography reconstruction of the fractured distal humerus. **D** The medial cannulated screw (arrow head) was positioned anterior to the ulnar nerve (arrow). **E**, **F** Postoperative radiographs obtained at final follow-up demonstrated bony union

In the single-plate-with-medial-screw group, the ulnar nerve was first identified. Unlike in the double-plate group, only in situ decompression was performed, and the ulnar nerve was not fully released from the ulnar groove. Fracture reduction was achieved and temporarily held with a K-wire. The lateral column was first stabilized with a VA-LCP posterolateral plate. When fixation of the distal fragment was deemed insufficient using the posterolateral plate, additional tension-band wiring was performed (Fig. 2). For medial column fixation, 4.0 or 6.5 mm cannulated screws (TCS, Stryker, Inc.) were inserted to provide interfragmentary compression and stability. When inserting the medial screw, it was positioned anterior to the ulnar nerve to avoid extensive medial and wide ulnar-nerve dissection (Fig. 3).

### Postoperative management

The postoperative rehabilitation protocol was identical for both groups. Postoperatively, the elbow joint was immobilized in a long-arm splint with the elbow at 90° of flexion for the first week. On postoperative days 5–7, the splint was removed, and a removable brace was applied. At this time, full flexion and extension were permitted with gentle, active-assisted exercises. One month after surgery, the patients were allowed to resume their usual daily activities.

### Data collection

Medical charts and radiographic images were retrospectively reviewed by two independent surgeons. Baseline demographic variables included age, sex, body mass index (BMI), and dominant hand. We obtained the time from injury to surgery, operative time, and postoperative visual analog scale (VAS) score. Postoperative VAS was specifically assessed 2 days following surgery to quantify early pain outcomes.

Bony union was evaluated at the final assessment using anteroposterior and lateral radiographs. Union was defined as the radiographic appearance of bridging callus across the fracture site in both projections. Elbow range of motion (flexion and extension) was measured, and functional outcomes were determined at the last follow-up using the Mayo Elbow Performance Score (MEPS).

Complications such as ulnar-nerve neuropathy, fracture-related infection, and reoperation were assessed at follow-up visits. Ulnar neuropathy was defined as sensory disturbance within the ulnar distribution or motor weakness of ulnar-nerve-innervated intrinsic muscles [11].

### Statistical analysis

The clinical efficacy and outcomes of double-plate fixation were statistically compared with those of single-plate fixation supplemented by a medial screw for distal humerus fractures. The distribution of continuous variables was examined using the Kolmogorov–Smirnov test to verify normality. For group comparisons, the Student’s *t*-test was applied to continuous variables, while categorical variables were analyzed using either the chi-squared test or Fisher’s exact test, depending on cell counts. Linear regression models incorporating continuous and dummy variables were employed to identify predictors of operative time and MEPS. Statistical analyses were conducted with SPSS software, version 28.0 (SPSS Inc., Chicago, IL, USA), and a *p* value < 0.05 was considered indicative of statistical significance.

## Results

### Patient characteristics

Of the 56 patients enrolled in this study, 30 were assigned to the double-plate group and 26 to the single-plate-with-medial-screw group. The mean age of the patients was 54.8 ± 19 (range, 17–85) years, and the mean follow-up duration was 18.2 ± 6.5 (range, 12–38) months. No significant differences were found between the groups in terms of demographic characteristics, such as age, sex, BMI, and AO/OTA classification. However, the time from injury to surgery was shorter in the double-plate group than in the single-plate-with-medial-screw group (Table 1).

**Table 1:** Demographic and fracture characteristics of the patients

Characteristics	Double plate (*n* = 30)	Single plate with a medial screw (*n* = 26)	*p*-Value
Age (years)	51.8 ± 19.3	58.3 ± 18.5	0.215
Sex (male:female)	7:23	6:20	1.000
BMI (kg/m^2^)	25.2 ± 2.7	24.2 ± 2.2	0.117
Dominant hand	14	14	0.789
Time from injury to surgery (days)	3.3 ± 2.63	5.2 ± 4.5	0.031
Follow-up (months)	16.7 ± 4.1	19.9 ± 8.2	0.077
AO/OTA classification			0.795
A2	15	12	
A3	15	14	

Values are presented as the mean ± standard deviation or *n* (%). *BMI* body mass index, *AO/OTA* Arbeitsgemeinschaft für Osteosynthesefragen/Orthopaedic Trauma Association

### Radiographic and clinical outcomes

All fractures achieved solid osseous union, as observed during follow-up. In two patients in the single-plate-with-medial-screw group, additional tension-band wiring was performed along with the posterolateral plate. No significant differences were observed between the two groups in terms of the postoperative pain scores (*p* = 0.293), range of flexion/extension (*p* = 0.185), or the MEPS (*p* = 0.194). However, the operative time was significantly shorter in the single-plate-with-medial-screw group than in the double-plate group (112.5 ± 25.7 min versus 172.2 ± 35.2 min, *p* < 0.05; Table 2). The final MEPS was not related to age, sex, BMI, dominant hand, AO/OTA fracture classification, time from injury to surgery, or fixation method. Similarly, the operative time was not associated with age, sex, BMI, dominant hand, AO/OTA fracture classification, or time from injury to surgery. However, regression analysis revealed that the operative time was associated with the fixation method (*p* < 0.05; Table 3).

**Table 2:** Surgical characteristics and outcomes of the patients

Characteristics	Double plate (*n* = 30)	Single plate with a medial screw (*n* = 26)	*p*-Value
Postoperative VAS score	4.3 ± 1.6	4.4 ± 1.3	0.293
Arc of flexion/extension	123.8 ± 8.3	126.5 ± 9.8	0.185
MEPS	89 ± 6.4	91.7 ± 7.1	0.194
Operative time (min)	172.2 ± 35.2	112.5 ± 25.7	< 0.05
Postoperative ulnar neuropathy	11	2	0.013
Reoperation	8	4	0.347

*VAS* visual analogue scale, *MEPS* Mayo Elbow Performance Score

**Table 3:** Linear regression analysis of the study variables

	Regression coefficient	*p*-Value
MEPS		
Age	0.057	0.307
Sex	0.686	0.774
BMI	−0.183	0.652
Dominant hand	1.774	0.359
AO/OTA classification	−2.946	0.13
Time from injury to surgery	−0.061	0.835
Fixation method	−2.299	0.25
Operative time		
Age	0.051	0.843
Sex	1.327	0.904
BMI	1.079	0.565
Dominant hand	−7.830	0.381
AO/OTA classification	11.639	0.195
Time from injury to surgery	−1.541	0.257
Fixation method	55.766	< 0.05

*MEPS* Mayo Elbow Performance Score, *BMI* body mass index, *AO/OTA* Arbeitsgemeinschaft für Osteosynthesefragen/Orthopaedic Trauma Association

### Complications

Postoperative ulnar neuropathy was the major complication observed after surgery (*n* = 13). Ulnar neuropathy occurred in 11 patients (37%) in the double-plate group and in two patients (8%) in the single-plate-with-medial-screw group (*p* = 0.013; Table 2). The majority of postoperative ulnar-nerve symptoms improved within 3 months without any treatment. However, one patient in the double-plate group experienced persistent ulnar-nerve symptoms requiring surgical treatment (ulnar neurolysis and submuscular anterior transposition), after which the patient fully recovered within 3 months. Apart from this case, 11 patients underwent implant removal due to implant-related discomfort. No significant differences in reoperation rates were observed between the two groups. In addition, no other complications, such as postoperative wound infection or implant failure resulting in non-union or malunion, were observed.

## Discussion

This retrospective comparative study demonstrated that single posterolateral plate fixation with a medial cannulated screw achieved clinical and radiographic outcomes comparable to those of the traditional double-plate fixation in extra-articular distal humerus fractures, while offering significant advantages by reducing operative time and ulnar nerve complications. The novel finding of this study was that strategically placing medial cannulated screws without extensive medial dissection maintained adequate fracture stability while substantially reducing nerve-related complications.

Our findings are consistent with previous studies examining alternative fixation methods for distal humerus fractures. Tejwani et al. demonstrated single locking plate fixation provides stability comparable to double-plate fixation in extra-articular distal humerus fractures [12]. Imatani et al. demonstrated successful outcomes with modified single-plate techniques, emphasizing the importance of adequate medial column support through alternative fixation methods [13]. The concept of medial-screw fixation was previously explored by Tanaka et al., who described a less invasive operative method using medial cannulated screws combined with single-plate fixation for transcondylar humeral fractures and reported favorable outcomes [9]. Their technique of positioning screws anterior to the ulnar nerve is similar to our approach and validates the biomechanical rationale for this technique. Kuwahara et al. also reported that posterolateral locked-plate and percutaneous medial-screw fixation for distal humerus fractures in older patients showed good functional outcomes and few complications [10]. However, several biomechanical and clinical studies have advocated for the superiority of double-plate fixation in distal humerus fractures [14, 15]. The discrepancies between our favorable outcomes and those reported in these biomechanical studies may be attributed to several factors. First, our study focused exclusively on extra-articular fractures, which may have biomechanical demands different from those of intra-aricular fractures examined in many comparative studies. Second, the in vivo healing biology and adaptive remodeling capacity may compensate for theoretical biomechanical disadvantages observed in laboratory testing.

The optimal intraoperative handling of the ulnar nerve—whether in situ release or anterior transposition—after fixation of distal humeral fractures remains controversial [1, 16–18]. Shin et al. found a 22% rate of postoperative ulnar-nerve palsies despite performing adequate release and nerve transposition in most patients. However, they reported that, when the nerve impinged upon the medial plate during elbow motion, irritation and transient sensory changes were developed [17]. Wiggers et al. identified risk factors for postoperative ulnar neuropathy, including age, sex, implant placement over or below the medial epicondyle, and the total number of surgeries [19]. They found that columnar fractures and the application of a medial plate were the only potential risk factors for iatrogenic postoperative ulnar neuropathy.

Although the optimal handling of the ulnar nerve is unclear, minimizing medial dissection of the distal humerus and ulnar-nerve mobilization are important for reducing the risk of postoperative ulnar neuropathy. The lower ulnar neuropathy rate in the single-plate-with-medial-screw group could be interpreted considering the difference in nerve management. The double-plate group required extensive mobilization of ulnar nerve [2, 7, 16, 20, 21], while the single-plate-with-medial-screw group required only in situ decompression when necessary. However, this reflects an inherent advantage rather than a confounding variable. The single-plate technique allows minimal medial dissection, as the medial screw can be inserted through a limited or percutaneous approach without extensive soft-tissue stripping. This minimally invasive approach naturally permits less nerve manipulation, reducing iatrogenic injury risk.

The high incidence of ulnar neuropathy associated with double-plate fixation in our study is consistent with that reported in the previous literature. Many previous studies have reported ulnar nerve complications, ranging from 15% to 40%, as a significant concern following traditional double-plate fixation [14, 20]. Pereles et al. specifically showed that medial-plate prominence and nerve irritation represent major postoperative concerns, particularly in older patients with thin soft-tissue coverage [22]. In contrast, the single plate with a medial cannulated screw preserves the natural anatomy around the ulnar nerve by avoiding extensive dissection and maintaining the nerve within its anatomical groove. Our ulnar neuropathy rate of 7.7% in the single-plate-with-medial-screw group compares favorably with other studies. Tanaka et al. reported that only 3.8% of patients treated with single-plate-and-medial-screw fixation experienced ulnar-nerve sensory symptoms, representing fewer complications compared with double-plate fixation [9]. Kuwahara et al. also reported that none of the 28 patients who underwent single-plate fixation with a medial screw for distal humerus fractures showed ulnar-nerve neuropathy [10]. The consistently low complication rates with medial-screw fixation may be attributed to minimal medial dissection and less extensive nerve manipulation compared with plate application, suggesting a nerve-protective advantage of this technique. In addition, avoiding complex medial-plate contouring and positioning reduced the operative time. Medial-plate application often requires meticulous anatomical reduction and precise screw trajectory, whereas cannulated-screw insertion can be performed more efficiently under direct visualization [23].

Our study had some limitations. First, we excluded patients with intra-articular distal humerus fractures because most previous biomechanical studies were performed using extra-articular distal humerus fracture models. In addition, we did not perform biomechanical testing to validate the mechanical adequacy of the single-plate construct. Thus, future studies are necessary to refine patient-selection criteria and treatment indications. Second, our study design represents a before-and-after comparative study, with the two fixation methods applied in different time periods. Additionally, the significant difference in time from injury to surgery between groups reflects this temporal change in institutional practice pattern. This sequential implementation introduces potential temporal bias and learning-curve effects that may have influenced our outcomes. The improved results in the single-plate-with-medial-screw-fixation group may be partially attributed to accumulated surgical experience rather than solely to the fixation technique itself. However, we found that the complication rate was significantly different depending on fixation method. In addition, all operations were performed by experienced elbow surgeons following the same perioperative protocol, and all data were analyzed by independent investigators, thus decreasing the potential for investigator bias.

## Conclusions

Both double-plate and single-plate-with-medial-screw fixation showed comparable union rates and functional outcomes in extra-articular distal humerus fractures. However, single-plate fixation with a medial screw required a shorter operative time and was associated with a lower incidence of postoperative ulnar neuropathy than double-plate fixation.

## Supplementary Information

Supplementary Material 1.

Supplementary Material 2.

## Data Availability

The datasets used and/or analyzed during the current study are available from the corresponding author upon reasonable request.
