# Supplementary material for: Clinical comparison of single posterolateral plate with medial-cannulated-screw fixation and double-plate fixation for extra-articular distal humerus fractures
Source: J Orthop Traumatol. 2026 May 1;27:32. doi: 10.1186/s10195-026-00918-6 (PMC13287183; doi:10.1186/s10195-026-00918-6)
Supplement: Supplementary file 1 — Supplementary Material 1. [file 10195_2026_918_MOESM1_ESM.pdf]

Chung-Ang University Hospital Institutional Review Board (IRB)
Tel :82-2-6299-2738 ~ 2741, FAX:82-2-6299-2860
102 Heukseok-ro Dongjak-Gu, Seoul 06973, Korea.

Official Report of IRB Review

This letter is to inform you of the results of IRB review.

**Reason for Request of review**	■ Initial Review ☐ Modifications or changes to the Protocol ☐ Unanticipated Problems or Non-Compliance ☐ Continuing Review ☐ Final Report ☐ Others		
**Title of Study**	Comparison between Single plate fixation with medial cannulated cancellous screw(CCS) fixation and double plate fixation for transcondylar fracture of distal humerus		
	**Protocol No.**	**Version No.**	1.0
**IRB No**	2506-026-19581	**Date of Review**	03. Jul. 2025
**Investigator**	Jae-Sung Lee M.D. Ph.D	**Department**	Department of Orthopaedic Surgery
**Study Agent(s)**	**Generic Names(s)**	**Brand Name(s)**	
**Phase**	☐ Phase I ☐ Phase II ☐ Phase III ☐ Phase IV ☐ PMS ☐ Bioequivalence ■ others		
**Proposed Period of Study**	03. Jul. 2025 ~ 02. Jul. 2026		
**Sponsor**			
**Result of Review**	■ Approved ☐ Approved with Condition ☐ Modifications Requires ☐ Disapproved		
**Continuing review report interval**	**1 year**		
**Reviewer's comments**	The initial document was approved.		

This is to certify that the information contained herein is true and correct as reflected in the records of the Chung-Ang University Hospital IRB. We certify that Chung-Ang University Hospital IRB is in full compliance with Good Clinical Practice as defined under the Korea Food and Drug Administration (KFDA) regulations and functions in accordance with the ICH GCP Guidelines (CPMP/ICH/135/95) and the Korean national ethics requirements.

Jeong-Kyu Lee
Chairperson

03-Jul-25
Date
