# Supplementary material for: Clinical comparison of single posterolateral plate with medial-cannulated-screw fixation and double-plate fixation for extra-articular distal humerus fractures
Source: J Orthop Traumatol. 2026 May 1;27:32. doi: 10.1186/s10195-026-00918-6 (PMC13287183; doi:10.1186/s10195-026-00918-6)
Supplement: Supplementary file 2 — Supplementary Material 2. [file 10195_2026_918_MOESM2_ESM.pdf]

Chung-Ang University Gwangmyeong Hospital
Institutional Review Board (IRB)

Tel: 82-2-2610-9224,9696, FAX: 82-2-2610-9220
110, Deokan-ro, Gwangmyeong-si, Gyeonggi-do, 14353, Korea.

Official Report of IRB Review

This is to inform that the following research has been reviewed and approved by Chung-Ang University Gwangmyeong Hospital, Institutional Review Board.

**IRB No**	2507-254-104			
**Protocol Title**	Comparison between Single plate fixation with medial cannulated cancellous screw(CCS) fixation and double plate fixation for transcondylar fracture of distal humerus			
	**Protocol No.**	-	**Version No.**	1.0
**Principal Investigator**	Jung, Hyoung Seok	**Department**	Orthopedic Surgery	
**Sponsor**	-			
**Type of Review**	■ Initial Review<br>□ safety information<br>□ Continuing Review<br>□ Final Report<br>□ Others	□ Modifications or changes to the Protocol<br>□ Unanticipated Problems or Non-Compliance<br>□ end of study report<br>□ cancellation of protocol		
**Contents of Review**	■ Protocol<br>(Version 1.0)<br>■ Case Report Form<br>■ Curriculum Vitae	□ Informed Consent Form<br>■ Certificate of Research ethics training<br>□ Budget<br>■ etc.		
**Result of Review**	■ Approved<br>□ Modifications Requires	□ Approved with Condition<br>□ Disapproved		
**Risk Level**	■ I	□ II	□ III	□ IV
**Approval Date**	27Jul2025	**Expiration Date**	26Jul2026	
**comment**	Review date: 27Jul2025 (Expedited review)			

This is to certify that the information contained herein is true and correct as reflected in the records of the Chung-Ang University Gwangmyeong Hospital IRB. We certify that Chung-Ang University Gwangmyeong Hospital IRB is in full compliance with Good Clinical Practice as defined under the The Ministry of Food and Drug Safety(MFDS) regulations and functions in accordance with the ICH-GCP Guidelines and the Korean national ethics requirements. Investigators of this research did not take part in the approval process.

Chung-Ang University Gwangmyeong Hospital
Institutional Review Board, Chair

ALL Chung-Ang University Gwangmyeong Hospital IRB APPROVED INVESTIGATORS MUST COMPLY WITH THE FOLLOWING:

1. Conduct the research as required by the protocol.
2. Use only the Consent Form approved by Chung-Ang University Gwangmyeong Hospital IRB.
3. Provide non-Korean speaking subjects with a certified translation of the approved Consent Form in the subject's first language. The translated version must be approved by the Chung-Ang University Gwangmyeong Hospital IRB.
4. Obtain pre-approval from the Chung-Ang University Gwangmyeong Hospital IRB of any changes in the research activity(except when necessary to protect human subjects; immediately report to the Chung-Ang University Gwangmyeong Hospital IRB any such emergency changes for the protection of human subjects).
5. Report to the Chung-Ang University Gwangmyeong Hospital IRB the death, hospitalization, or serious illness of any study subject.
6. Promptly report to the Chung-Ang University Gwangmyeong Hospital IRB any new information that may adversely affect the safety of the subjects or the conduct of the trial.
7. Provide reports to the Chung-Ang University Gwangmyeong Hospital IRB concerning the progress of the research, when requested.
8. Obtain pre-approval of study advertisements from the Chung-Ang University Gwangmyeong Hospital IRB before use.
9. Conduct the informed consent process without coercion or undue influence, and provide the potential subject sufficient opportunity to consider whether or not to participate.
